# The oncoprotein TBX3 is controlling severity in experimental arthritis

**DOI:** 10.1186/s13075-018-1797-3

**Published:** 2019-01-10

**Authors:** Samra Sardar, Alish Kerr, Daniëlle Vaartjes, Emilie Riis Moltved, Edita Karosiene, Ramneek Gupta, Åsa Andersson

**Affiliations:** 1 Section for Molecular and Cellular Pharmacology, Department of Drug Design and Pharmacology, University of Copenhagen, Copenhagen, Denmark; 2 Present address: Nordic Bioscience A/S, Copenhagen, Denmark; 3 Present address: Nuritas, Dublin, Ireland; 4 Present address: Division of Medical Inflammation Research, Department of Medical Biochemistry and Biophysics, Karolinska Institutet, Stockholm, Sweden; 5 Present address: IQVIA, Copenhagen, Denmark Denmark; 6 Department of Bio and Health Informatics, Kemitorvet 208, Technical University of Denmark, Lyngby, Denmark; 7 Present address: Novo Nordisk A/S, Copenhagen, Denmark; 8 Rydberg Laboratory of Applied Sciences, ETN, Halmstad University, Halmstad, Sweden

**Keywords:** Collagen-induced arthritis, Transcriptional regulation, TBX3, TBX5, Biomarker, Eae39r

## Abstract

**Background:**

Development of autoimmune diseases is the result of a complex interplay between hereditary and environmental factors, with multiple genes contributing to the pathogenesis in human disease and in experimental models for disease. The T-box protein 3 is a transcriptional repressor essential during early embryonic development, in the formation of bone and additional organ systems, and in tumorigenesis.

**Methods:**

With the aim to find novel genes important for autoimmune inflammation, we have performed genetic studies of collagen-induced arthritis (CIA), a mouse experimental model for rheumatoid arthritis.

**Results:**

We showed that a small genetic fragment on mouse chromosome 5, including *Tbx3* and three additional protein-coding genes, is linked to severe arthritis and high titers of anti-collagen antibodies. Gene expression studies have revealed differential expression of *Tbx3* in B cells, where low expression was accompanied by a higher B cell response upon B cell receptor stimulation in vitro. Furthermore, we showed that serum TBX3 levels rise concomitantly with increasing severity of CIA.

**Conclusions:**

From these results, we suggest that TBX3 is a novel factor important for the regulation of gene transcription in the immune system and that genetic polymorphisms, resulting in lower expression of *Tbx3*, are contributing to a more severe form of CIA and high titers of autoantibodies. We also propose TBX3 as a putative diagnostic biomarker for rheumatoid arthritis.

**Electronic supplementary material:**

The online version of this article (10.1186/s13075-018-1797-3) contains supplementary material, which is available to authorized users.

## Background

Rheumatoid arthritis (RA), affecting 0.5–1% of the world’s population, is characterized by chronic inflammation, which primarily affects the synovial joints leading to tissue damage and physical disability. Furthermore, the consequences of immunological abnormalities and systemic inflammation may lead to premature mortality. The disease follows a heterogeneous clinical course with a gender bias towards women. The etiology mainly remains unknown due to the multifactorial nature and complexity of the disease. Both genetic and environmental factors are reported to play a role in the predisposition to RA [1]. Genes within the major histocompatibility complex (MHC), also called the human leukocyte antigen (HLA) complex, are the strongest genetic factors for RA [2]. In addition, a large number of non-MHC genes have been shown to contribute to RA pathogenesis [3, 4].

Despite the introduction of new, effective therapies for RA, not all patients respond to the available treatments. Therefore, there is an unmet need for novel drugs and better biomarkers that can improve early detection, determine prognosis and disease activity, stratify patients for various therapeutic options, and monitor the response to therapy [5]. Novel biomarkers should be robust in their association with disease activity and offer predictive value in selection of appropriate therapy.

Experimental animal models, providing a genetically homogeneous and well-controlled experimental environment, are invaluable tools in genetic studies and drug and biomarker discovery in RA, whereby collagen-induced arthritis (CIA) is the most commonly used model [6]. After immunization with bovine collagen type II (CII), the B10.RIII mouse strain develops CIA, whereas the MHC-matched RIIIS/J mouse strain is resistant to arthritis development. The two particular mouse strains are therefore suitable for studies of non-MHC genes involved in the development of CIA [7, 8]. A strategy involving gene-segregating crosses between the two mouse strains, and development of congenic mice, allows for identification of novel non-MHC candidate genes and related disease pathways [9].

The genetic locus *Eae39*, located on mouse chromosome 5, was previously linked to experimental autoimmune encephalomyelitis (EAE), an experimental model for multiple sclerosis. Subsequently, it was also shown to influence CIA development and severity [8, 10]. *Eae39* congenic and sub-congenic lines were bred on the genetic background of the B10.RIII strain [7]. In the present study, the *Eae39* sub-locus, *Eae39r,* was investigated. This locus comprises four protein-coding genes: Mediator complex subunit 13-like (*Med13l*), T-box transcription factor 3 (*Tbx3*), T-box transcription factor 5 (*Tbx5*), and Probable RNA-binding protein 19 (*Rbm19*). The MED13L protein is part of the cyclin-dependent kinase (CDK)-8 module of the mediator complex and acts as a key regulator of RNA polymerase II-dependent gene transcription [11, 12]. The *Rbm19* gene encodes for a protein contributing to ribosomal (r) RNA processing, a key step in ribosome biogenesis [13]. It belongs to a group of RNA binding proteins (RBPs) that have been associated with neurological disorders, cancers and inflammatory diseases [14]. However, the exact function and role of *Rbm19* in disease associations have not been well-studied.

The T-box genes *Tbx3* and *Tbx5* are closely linked and share 98% and 96% homology between mouse and human, respectively [15]. TBX5 acts as a transcription activator [16], whereas TBX3 functions predominantly as a transcriptional repressor. Nevertheless, TBX3 contains both activation and repression domains that are suggested to be modulated in different cellular milieus [17, 18]. Among other functions, both proteins are involved in bone development [18–20] and remodeling [21]. Mutations in the human *TBX3* and *TBX5* genes cause ulnar-mammary syndrome (UMS, OMIM 181450) and Holt-Oram syndrome (HOS, OMIM 142900), respectively. Both syndromes cause defects in limb development [16, 22], which asserts their link to bone pathways recently recognized to interact with immune pathways [23]. However, the role of the TBX3 and TBX5 proteins in the immune system, their link to RA, and their biomarker potential, remains unexplored.

With the aim to elucidate the role of the *Eae39r* genetic locus in autoimmune inflammation, we investigated CIA pathogenesis in B10.RIIIS/J-*Eae39r* congenic and sub-congenic mice, followed by studies of polymorphisms, gene transcription, and protein expression. We report that mice with allelic variants in the upstream and downstream regions of the *Tbx3*, *Tbx5*, and *Rbm19* genes, develop more severe CIA compared to littermate controls. In addition, these mice develop significantly enhanced anti-CII antibody responses and have altered B cell response upon activation in vitro. In the present study, we identified reduced expression of *Tbx3* in the spleens of sub-congenic mice and showed that activation of B cells is concomitant with a changed level of active TBX3 protein. This study gives novel insight into CIA candidate genes and provides the first evidence for TBX3 as an RA candidate gene and putative RA biomarker, thereby furthering our understanding of the disease pathogenesis.

## Methods

### Mice

BR.RIIIS/J-*Eae39r* congenic mice were produced by introduction of the *Eae39r* fragment from the CIA-resistant RIIIS/J donor strain, purchased from Jackson Laboratory (Bar Harbor, ME, USA), to the CIA susceptible B10.RIII background strain, provided by J. Klein (Tübingen, Germany), as previously described [8]. The sub-congenic line BR.RIIIS/J-*Eae39r2* was produced by further inter-crossing heterozygous BR.RIIIS/J-*Eae39r* mice. All mice were kept and bred under standard conditions in the animal facility at the Department of Drug Design and Pharmacology, Faculty of Health and Medical Sciences, University of Copenhagen, Denmark. The Danish Animal Experiment Inspectorate license number is 2010/561–1920 and 2015-15-0201-00794.

### Genotyping

Genomic DNA (gDNA) was purified from mouse ear biopsies with High Pure PCR template preparation kit (11796828001; Roche Holding AG, Basel, Switzerland) according to the manufacturer’s protocol. Purified gDNA was used for genotyping by high-resolution melting (HRM) SNP genotyping for rs33583463 (5:118596773 bp; mouse genome assembly GRCm38) and rs29824716 (5:120043597 bp; mouse genome assembly GRCm38), and by PCR-agarose gel electrophoresis method for the homemade microsatellite marker D5tbxhm17 (5:119660373 bp; mouse genome assembly GRCm38). HRM analysis was carried out on the Roche LightCycler 480 using High Resolution Melting Master (04909631001, Roche Holding AG, Basel, Switzerland) as described elsewhere [26]. Microsatellite marker genotyping was determined by analyzing PCR products on a MegaBACE1000 genotyping system (Amersham Biosciences, Little Chalfont, UK) or on a 3% agarose gel as previously described [9]. Primer sequences are provided in the Additional file 1: Table S1. Based on genotype, mice were allocated into homozygous congenic, sub-congenic or wild type control groups for various experiments.

### Induction and evaluation of CIA

CIA was induced in 8–10 weeks old male mice by intra-dermal injection of 100 μg bovine CII (CII-7806; Sigma-Aldrich, St Louis, MO, USA) emulsified in incomplete Freund’s adjuvant (IFA) (F5506; Sigma-Aldrich, St Louis, MO, USA) at the base of the tail (day 0) followed by a booster dose of 50 μg CII emulsified in IFA on day 35. Clinical disease was monitored in a blinded manner three times a week, whereby arthritis was evaluated based on inflammation (erythema and swelling) of affected joints. Each mouse received a score according to a scoring system whereby each inflamed toe (first phalanx excluded), all inflamed knuckles and inflamed wrist or ankle were assigned one point giving a maximum of 6 points per paw and a theoretical maximum score of 24 per mouse. According to the Danish Animal Experiment Inspectorate approved humane endpoints, mice receiving scores above 10 were euthanized. The experiment was terminated when no mice (irrespective of group) developed arthritis in previously non-affected paws on three consecutive observations or when the groups were left too small for adequate statistical power.

For comparison of CIA development between congenic, sub-congenic and control groups, arthritis scores for mice belonging to a particular genotype were analyzed together in order to calculate mean score versus days after immunization (disease progression), mean maximum score (disease severity), incidence, ethical survival, mean day of onset, and area under the curve (AUC; the sum of scores during the course of the disease).

A total of 24 male B10.RIII mice were used for the time-course CIA study, whereby 20 mice were immunized and 4 were left unimmunized. CIA was induced on day 0, by subcutaneous immunization with 100 μg CII emulsified in IFA containing 50 μg *Mycobacterium tuberculosis* H37Ra (DIFCO laboratories, Detroit, MI, USA) and boosted on day 35 with 50 μg CII in IFA. CIA was scored as aforementioned and four mice were sacrificed on different days of the experiment, covering pre-clinical (day 7, 15, and 25), onset/progressive (day 42) and late (day 68) phases of CIA. Blood was collected for serum analysis, paws were snap-frozen in liquid nitrogen for later analysis and spleen was used for isolating CD19^+^ B cells for DNA activity assay.

### Enzyme-linked immunosorbent assay (ELISA)

For anti-CII ELISA, serum was prepared from blood collected on days 0 and 15 (by submandibular bleeding), and on the last day of CIA (by cardiac puncture). The levels of CII-specific IgM, IgG_1_, IgG_2c_, and IgG_3_ antibodies were determined as previously described [8]. Briefly, serum dilutions were applied to 96-well micro-titer plates (Nunc maxisorp, Roskilde, Denmark) coated overnight with CII in PBS (0.5 μg/well) and subsequently blocked with 1% BSA/PBS solution. The antigen-antibody binding signal was revealed by biotinylated secondary antibodies: goat anti-mouse IgM (1020–08), anti-IgG_1_ (1070–08), anti-IgG_2c_ (1079–08), and anti-IgG_3_ (1100–08) (SouthernBiotech, Birmingham, AL, USA) and Avidin-HRP (554058, BD Pharmingen, San Jose, CA, USA), followed by detection with 2,2′-Azinobis [3-ethylbenzothiazoline-6-sulfonic acid]-diammonium salt (ABTS) substrate (A1888; Sigma-Aldrich, St Louis, MO, USA). A SpectraMax Microplate Reader (Molecular Devices Corporation, Sunnyvale, CA, USA) was used to read the absorbance at a wavelength of 405 nm with a wavelength correction set to 492 nm. Pooled serum from arthritic mice were used as a standard and the antibody levels were measured as arbitrary concentrations.

For interleukin-2 (IL-2) cytokine ELISA, supernatants from triplicate CD4^+^ T lymphocyte cultures were collected at 48 h and analyzed by standard protocol for sandwich ELISA described elsewhere (BD Biosciences, Immune function analysis application handbook).

### Sequencing and bioinformatics analysis

**Fig. 1:**
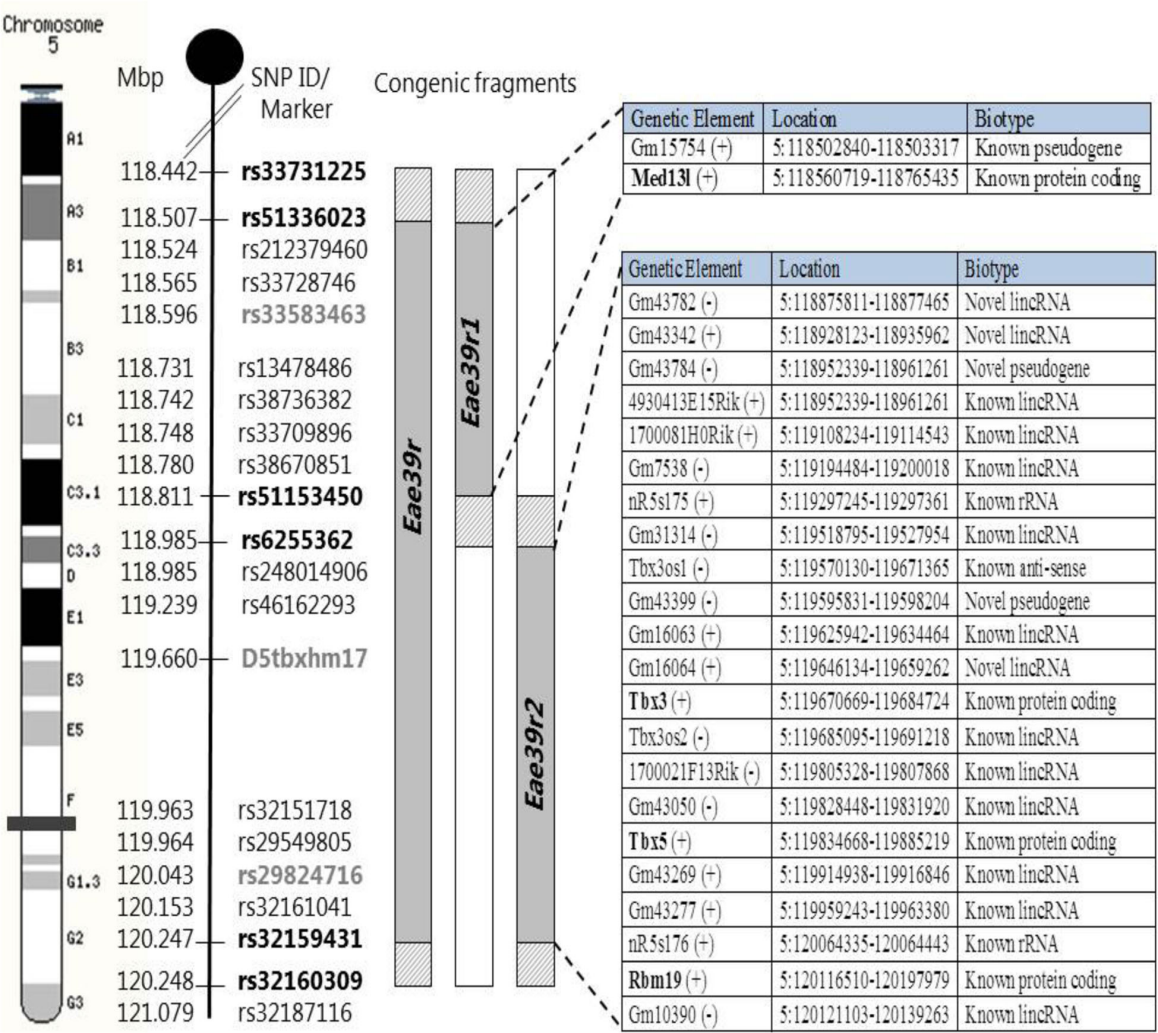
Markers defining the location of the *Eae39r* locus, and the *Eae39r1* and *Eae39r2* sub-loci on mouse chromosome 5, and the genetic elements included in the *Eae39r* region. SNP Ids in bold (black) represent the borders of the congenic and sub-congenic fragments. The SNP Ids/microsatellite markers shown in gray were used for genotyping. Solid gray represents RIIIS/J alleles bred on to the B10.RIII background (white), while the diagonally lined gray represents regions of unknown genotype. The genetic elements defining the *Eae39r* fragment are shown in tabular form along with their location and biotype (according to Ensembl release 74) and protein coding genes are bolded. Mbp, mega base pair; SNP, single nucleotide polymorphism; lincRNA, long intervening noncoding RNA; rRNA, ribosomal RNA. (+) = forward strand, (−) = reverse strand

The borders of the congenic and sub-congenic fragments were mapped using the aforementioned genotyping markers, spanning the *Eae39r* fragment, and were fine-mapped by sequencing the genetic regions around those borders by Sanger sequencing (GATC biotech, Germany). The genomic locations of the borders and of single nucleotide polymorphisms (SNPs) used for fine-mapping are shown in Fig. 1. Using the defined borders, genetic elements located in the congenic and sub-congenic fragments, their biotype [27] and expression details [28] were determined, followed by literature mining. PubMed [29] and Google Scholar [30] databases were searched using the keywords collagen-induced arthritis, rheumatoid arthritis, autoimmunity, inflammation, lymphocytes, and cell signaling. The retrieved information was used to prioritize the genes for further studies.

To identify genetic variations in protein coding genes located in the *Eae39r2* sub-congenic fragment, Sanger sequencing (GATC biotech, Germany), followed by computational analysis, was performed. All coding exons and regulatory regions of the *Tbx3* and *Tbx5* genes, including promoters (2 kb upstream of transcriptional start site), 5’ UTR, and 3’ UTR, in addition to regions containing documented non-synonymous SNPs for the *Rbm19* gene were sequenced (primers are listed in Additional file 1: Table S2). The genomic locations of these regions, based on mouse genome build GRCm38, were retrieved from Ensembl [27].

The identified variants were classed based on their relationship to genes in the dbSNP [31] and Ensembl [27] databases. Furthermore, web-based prediction tools were used to check for possible effect of intronic and 5’/3’UTR variants on transcription factor binding [32, 33] and micro RNA (miRNA) binding [34], respectively. For prediction of transcription factor binding site (TFBS) 20 nt region around the confirmed SNPs, between B10.RIII and B10.RIIIS/J-*Eae39r2* mice, was checked for difference in binding using two prediction tools, CONSITE [32] and PROMO [33]. The disruption/introduction of TFBS was reported with respect to background strain i.e. B10.RIII.

### Isolation of B and T lymphocytes

Mouse spleens were harvested and mashed through a 40-μm cell strainer to prepare single-cell suspensions followed by depletion of red blood cells using BD Lysing Buffer (555899; BD Biosciences, San Jose, CA, USA). CD19^+^ B lymphocytes for cell proliferation assay, real time PCR, and TBX3 activity assay, were positively selected from splenic cell suspensions by using the EasySep™ CD19 selection kit (18954; Stemcell Technologies, Vancouver, Canada) according to the manufacturer’s recommended protocol. Pan T lymphocytes for real time PCR and CD4^+^ T lymphocytes for cell proliferation assay were isolated from the CD19^−^ flow-through using EasySep™ T cell isolation kit (19851; Stemcell Technologies, Vancouver, Canada) and EasySep™ CD4^+^ T cell enrichment kit (19752; Stemcell Technologies, Vancouver, Canada), respectively. Cell purity was > 90% for CD19^+^ B cells and > 92% for pan T and CD4^+^ T cells, as determined by flow cytometry.

### Cell cultures, stimulation, and proliferation assay

Purified B lymphocytes were plated at 3 × 10^5^ per well in round-bottomed 96-well cell culture plates (Corning, New York, USA). The cells were cultured in complete Dulbecco’s modified eagle’s medium (DMEM) GlutaMAX-I (61965026), 5% heat-inactivated fetal bovine serum (FBS- 10082139), 1 mM HEPES (15630056), 50 μM 2-Mercaptoethanol (31350010), and 1% penicillin/streptomycin (10378016; Invitrogen, Thermo Fisher Scientific, Waltham, MA, USA), alone or in the presence of 0–10 μg/ml lipopolysaccharide (LPS) (L2880; Sigma-Aldrich, St Louis, MO, USA), 0–40 μg/ml goat anti-mouse IgM F (ab’)_2_ (115–006-075; Jackson ImmunoResearch Laboratories, Inc. Baltimore, PA, USA) or a combination of 0–4 μg/ml anti-mouse CD40 antibody, clone 1C10 (14–0401; eBioscience, San Diego, CA, USA) and 10 ng/ml mouse IL-4 recombinant protein (14–8041; eBioscience, San Diego, CA, USA). For CD4^+^ T cells, 10^5^ purified cells per well were stimulated with plate-bound purified anti-mouse CD3 antibody, 0-3 μg/ml, clone 145-2C11 (550275; BD Biosciences, San Jose, CA, USA) and anti-mouse CD28 antibody, fixed at 3 μg/ml, clone 37.51 (16–0281; eBioscience, San Diego, CA, USA). After culturing for 48 h at 37 °C in 5% CO_2_, the cells were pulsed for 16–18 h with 1 μCi per well ^3^H-thymidine (Perkin Elmer, Waltham, MA, USA), and incorporation was measured on a TopCount Scintillation Counter (Perkin Elmer, Waltham, MA, USA) as counts per minute (cpm).

For the time-course CIA, purified B cells from CII immunized mice, at the indicated time points, were cultured for 48 h in complete DMEM 5% FCS at 37 °C, 5% CO_2_ with or without stimulation with a fixed concentration of anti-mouse IgM F (ab’)_2_ (10 μg/ml).

### Flow cytometric analysis

Single-cell suspensions of spleen tissue were prepared in complete DMEM as described above: 1 × 10^6^ cells were pre-incubated with 1 μg of Fc receptor block (anti-CD16/CD32 antibody clone 93; 14–0161-81, eBioscience, San Diego, CA), prior to staining with 0.1 μg of antibodies for 15 min on ice. Rat anti-mouse CD19 fluorescein isothiocyanate (anti CD19-FITC) antibody clone 1D3 (553785), anti-mouse CD4-phycoerytherin (anti CD4-PE) antibody clone GK1.5 (553730), and anti-mouse CD8-phycoerytherin-cyanine 5 (anti CD8-PE-Cy5) antibody clone RPA-T8 (555368), all purchased from BD Biosciences (San Jose, CA, USA) were used. The stained cells were analyzed using Gallios Flow Cytometer (Beckman Coulter, Brea, CA, USA) and FlowJo software (Tree Star Inc., Ashland, OR, USA).

### Reverse transcriptase-polymerase chain reaction (RT-PCR)

For transcript quantification, snap-frozen whole ankle joints (including synovium, adjacent tissues, and bones) were pulverized using a mortar and pestle, followed by homogenization with IKA T-10 Ultra-Turrax homogenizer (IKA-Works, Staufen im Breisgau, Germany). Snap-frozen spleen tissue was also homogenized with IKA T-10 Ultra-Turrax homogenizer. Total RNA was extracted from joint and spleen homogenates using the RNeasy Plus Universal Mini Kit (73404; Qiagen, Hilden, Germany) and from purified splenic B and T lymphocytes using the RNeasy Micro Kit (74034; Qiagen, Hilden, Germany) followed by removal of gDNA remnants by Turbo DNA free kit (AM1907; Invitrogen, Carlsbad, CA, USA) according to the manufacturer’s protocol. The concentration and purity of total RNA was measured on the NanoDrop 2000 (Thermo Fisher Scientific, Waltham, MA, USA), while integrity was verified on a 1% agarose gel. Complementary DNA (cDNA) was synthesized from 1 μg of extracted RNA using Superscript III First Strand Synthesis Kit (11752–250; Invitrogen, Carlsbad, CA, USA) and SYBR green-based real time PCR was performed with PrecisionPlus qPCR Mastermix (Primerdesign Ltd., Eastleigh, UK) and LightCycler480 Real-Time PCR System (Roche, Mannheim, Germany). Relative quantification was performed by calculating 2^^^(−delta delta CT) (2^-ΔΔ*CT*^), whereby ΔΔCT was taken as the difference in normalized crossing threshold (CT) values between experimental (sub-congenic) and control samples, and the CT values of both groups were normalized to *Ubiquitin C* (*Ubc*) as a housekeeping gene. Primer sequences are listed in Additional file 1: Table S3.

### DNA binding activity assay for TBX3

TBX3 activity was measured in the nuclear lysate of unstimulated and B cell receptor (BCR)-stimulated CD19^+^ B cells, and joint tissue homogenates using pre-validated TFact™ DNA binding ELISA kit (TFE-7159; Assay Biotechnology, Sunnyvale, CA, USA). The nuclear extraction was performed with few changes while the immunoassay was done according to the manufacturer’s protocol. Briefly, the cells were lysed on ice for 15 min in a hypo-osmotic lysis buffer (10 mM HEPES, 10 mM KCl, 1.5 mM MgCl_2_, and 0.1 mM DTT) containing protease and phosphatase inhibitors-PPI (05892791001; Roche Diagnostics GmbH, Mannheim, Germany). The nuclear proteins were then extracted in the provided nuclear extraction buffer with PPI by incubating the samples on ice for 40 min and vortexing intermittently. A Pierce BCA Protein Assay (23227; Thermo Fisher Scientific, Waltham, MA, USA) was employed to determine the amount of nuclear proteins in all samples using known concentrations of BSA as standards.

The extracted nuclear samples were applied to the wells pre-coated with oligonucleotides specific for binding TBX3 and incubated overnight at 4 °C. Following incubation with TBX3 primary antibody (for 2 h), and HRP-conjugated secondary antibody (for 1 h), 3, 3′, 5, 5’-tetramethylbenzidine (TMB) substrate was added to allow color development, which was quenched after 20 min by addition of stop solution. Absorbance of each well was determined at a wavelength of 450 nm (correction set to 540 nm) using a SpectraMax Microplate Reader (Molecular Devices Corporation, Sunnyvale, CA, USA). Active TBX3 levels were measured as a relative concentration in comparison to standard curve of nuclear lysate positive control (NLPC) provided in the assay kit.

### Serum quantification of TBX3

TBX3 was quantitated in serum samples, from the time-course CIA study, using a pre-validated mouse TBX3 ELISA Kit (MBS2886293; MyBioSource, Inc. San Diego, CA, USA). Standard sandwich ELISA was performed according to the manufacturer’s protocol with few changes in incubation time and temperature to reduce the background signal. Briefly, 100 μl of appropriate dilutions of TBX3 standard (0–2.5 ng/ml) and samples were applied per well to the 96-well microtiter plate pre-coated with TBX3 capture antibody. The plate was incubated overnight at 4 °C followed by addition of the recommended amount of biotin-conjugated detection antibody (detection reagent A). After 2 h incubation at room temperature and washing steps, avidin-HRP (detection reagent B) was added at room temperature for 1 h. TMB substrate was added to allow color development, which was quenched after 20 min by addition of 0.1 M H_2_SO_4_ stop solution. Absorbance of each well was determined at a wavelength of 450 nm (correction set to 540 nm) using a SpectraMax spectrophotometer. The sensitivity limit of the assay was 0.091 ng/ml, and the inter-assay and intra-assay coefficient of variation (CV) was ≤ 10%, as reported by the manufacturer.

### Statistical analysis

Statistical analyses were performed using GraphPad Prism 7.03. All data on CIA (except disease incidence and ethical survival data), ELISA, in vitro proliferation, and TBX3 activity were analyzed using the Mann-Whitney U test. The chi-square test and Fischer’s exact test were applied for analysis of CIA incidence and ethical survival data, respectively. RT-PCR data were analyzed by two-way Student’s *t* test. *P*-values less than 0.05 were considered significant in all cases.

## Results

### The *Eae39r2* locus controls arthritis development and anti-CII antibody response

**Fig. 2:**
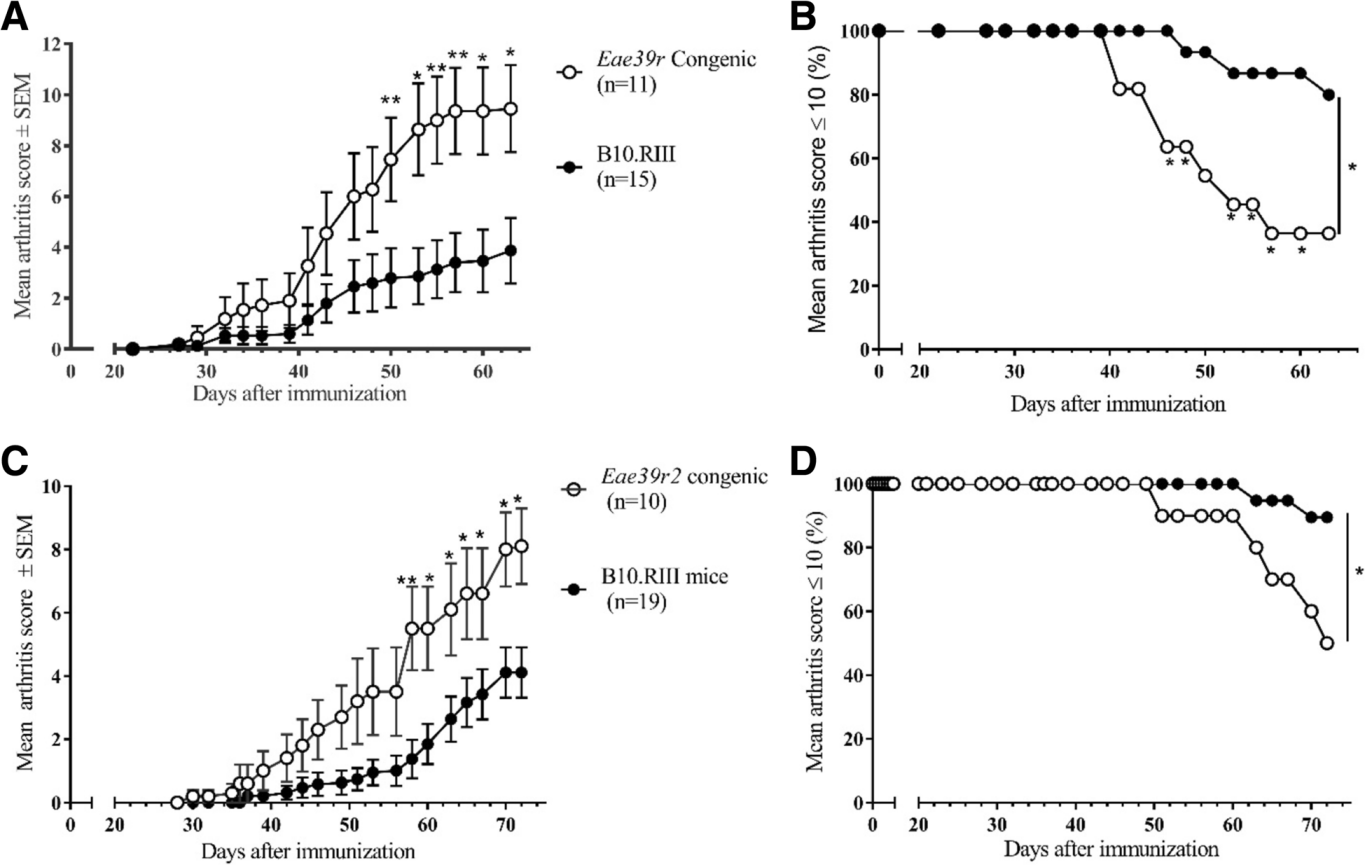
Collagen-induced arthritis (CIA) development in B10.RIIIS/J-*Eae39r* and B10.RIIIS/J-*Eae39r2* congenic and sub-congenic mice in comparison to littermate controls. **a** CIA progression in B10.RIIIS/J-*Eae39r* congenic mice represented as mean arthritis score for each group ± standard error of the mean (SEM). **b** Ethical survival curve for B10.RIIIS/J-*Eae39r* congenic mice - based on the animal healthcare inspectorate guidelines that mice attaining a total score > 10 should be euthanized. **c** CIA progression and **d** ethical survival in B10.RIIIS/J-*Eae39r2* sub-congenic mice. Data were analyzed using the Mann-Whitney U test for disease progression and the chi-squared test for ethical survival; *p < 0.05, **p < 0.01

In order to further dissect the genetics of the previously reported non-MHC locus *Eae39r* [8, 35] and its impact on CIA in mice, we established an B10.RIIIS/J-*Eae39r* congenic mouse line, carrying the *Eae39r* fragment from the CIA-resistant RIIIS/J (H-2^r^) strain on the background genome of the CIA-susceptible B10.RIII (H-2^r^) strain, and two *Eae39r* sub-congenic mouse lines: B10.RIIIS/J-*Eae39r1* and B10.RIIIS/J-*Eae39r2* (Fig. 1). Arthritis development was studied in the B10.RIIIS/J-*Eae39r* congenic and B10.RIIIS/J-*Eae39r2* sub-congenic mice by immunization with bovine CII and the results from the clinical arthritis scoring showed that B10.RIIIS/J-*Eae39r* congenic and B10.RIIIS/J-*Eae39r2* sub-congenic mice developed significantly more severe arthritis compared to their littermate control mice (Fig. 2a-d and Table 1). These results show that the *Eae39r2* locus harbors genes that control the development of CIA. Similar results were obtained in a second independent CIA study comparing the B10.RIIIS/J-*Eae39r2* sub-congenic and control mice (data not shown).

**Table 1:** Collagen-induced arthritis (CIA) phenotypes in *Eae39r* congenic and *Eae39r2* sub-congenic mice

Disease parameter (phenotype)	Experimental group					
	*Eae39r* congenic (*n* = 11)	Littermate controls (*n* = 15)	*p* value^a^	*Eae39r2* sub-congenic (*n* = 10)	Littermate controls (*n* = 19)	*p* value^a^
Incidence	9/11 (82%)	8/15 (53%)	0.2167	10/10 (100%)	15/19 (79%)	0.2680
Mean max score^b^	9.7 ± 1.6	3.9 ± 1.3	0.0041**	8.30 ± 1.14	4.4 ± 0.8	0.0118*
AUC^c^	80.8 ± 18	29.7 ± 11.2	0.0120*	67.8 ± 17.6	25.7 ± 7.2	0.0134*
Mean day of disease onset	42.2 ± 2.6	36.3 ± 3.4	0.1942	47.9 ± 3.4	53.6 ± 2.9	0.2529
Number of mice euthanized^d^	7/11 (64%)	3/15 (20%)	0.0426*	5/10 (50%)	2/19 (10.5%)	0.0302*

^a^Data on the incidence and number of mice euthanized were analyzed using the chi-squared test and the other variables were analyzed using the Mann-Whitney U test; *p < 0.05, **p < 0.01

^b^Mean max score, indicating collagen-induced arthritis severity, is the mean (± SEM) of the maximum score for all affected mice in the group

^c^Area under curve (AUC) is the mean (± SEM) of the sum of scores for mice of the corresponding genotype (days 22–63 for *Eae39r* and days 30–72 for *Eae39r2*)

^d^Mice having a score above 10 were euthanized during the experiment as per recommendations of animal welfare authorities

The disease-enhancing effect from the *Eae39r2* genetic fragment was also accompanied by significantly higher titers of anti-CII antibodies, compared with littermate controls, in both early (day 15) and late (day 72) phases of disease (Table 2). This shows that genes in this region, in addition, control antibody responses to CII, which constitute a major part of the CIA pathogenesis [36].

**Table 2:** Anti-collagen type II antibody levels in serum of *Eae39r2* sub-congenic mice

Congenic fragment	Days after collagen type II immunization	Antibody isotype	Antibody titers in groups of mice (AU/ml)^a^		Statistics^b^
			Homozygous Congenic	Littermate controls	*p* value
*Eae39r2*	15 (preclinical phase)	IgG_1_	252 ± 46	96 ± 26	0.0041**
		IgG_2C_	148 ± 11	77 ± 10	< 0.0001****
		IgG_3_	7 ± 0.5	4 ± 0.3	0.0067**
		IgM	55 ± 3	37 ± 4	0.0002***
	72 (clinical phase)	IgG_1_	5044 ± 1363	1852 ± 486	0.0002***
		IgG_2C_	822 ± 113	323 ± 45	< 0.0001****
		IgG_3_	9 ± 0.5	5 ± 0.4	< 0.0001****
		IgM	11 ± 4	9 ± 3	0.144

^a^Arbitrary units per ml (AU/ml) are calculated in comparison to a standard curve of pooled serum from arthritic mice in the same experiment

^b^Data were analyzed using the Mann-Whitney U test; *p < 0.05, **p < 0.01, ***p < 0.001

### *Tbx3* and *Tbx5* are candidate genes in the *Eae39r2* locus

Sanger sequencing of documented SNPs from mouse genome databases, in and around the *Eae39r* locus, allowed the determination of borders of the congenic and sub-congenic fragments as closely as possible, thereby narrowing down the list of genes that could be responsible for the CIA-enhancing effect. The *Eae39r* fragment spanned over 1.8 Mbp, between rs33731225 (5:118442102 bp) and rs32160309 (5:120248221 bp); and *Eae39r2* was defined to be 1.4 Mbp, between rs51153450 (5:118811970 bp) and rs32160309 (5:120248221 bp) (Fig. 1). Since *Eae39r2* was the smallest fragment controlling CIA development, further focus of this study was restricted to this fragment. Using online resources, all protein-coding and non-protein-coding genetic elements located within the *Eae39r2* locus were listed (Table 3).

**Table 3:** Genetic elements located in the *Eae39r2* region, their expression, and literature overview

Genetic Element^a^	Expression^b^	Literature summary
***Tbx3*** *(+)*	Expressed in **colon, lung, heart,** liver, adrenal gland and spleen	Controls cell proliferation and differentiation [37, 60, 62]; enhances tumor invasiveness [18]; regulates gene splicing [68]; modulates number of cell signaling pathways involved in the development of limb, mammary gland, and heart [18]; regulates bone formation [69, 70] and bone resorption [71]; downregulated in the early phase of collagen-induced arthritis (CIA) [21]; linked to rheumatoid arthritis (RA) in a genome-wide association study (GWAS) [72]; and modulated in RA twin lymphoblastoid B cell lines compared with healthy co-twin LCLs [73].
***Tbx5*** *(+)*	Expressed in **heart, lung, bone,** and urinary bladder	Controls cell proliferation, differentiation and migration [74, 75]; involved in cell signaling pathways [76–78]; regulates bone growth and maturation [20, 79] and system development [80, 81]; downregulated in the early phase of CIA [21]; linked to osteochondrosis in GWAS [82] and RA by activation of RA synovial fibroblasts [83].
***Rbm19*** *(+)*	Expressed in **heart, lung, uterus,** and skeletal muscle	Key role in synthesis of the small eukaryotic ribosome subunit [84], pre-implantation development [13], and mammary gland morphology [85].
*1700081H04Rik (+)*	Expressed in **testis**, brain, heart, kidney and liver	No data available
*n-R5s175 (+)*	Expressed in **kidney**, **liver**, testis, brain, spleen, skeletal tissue, and lung	No data available
*Tbx3os1 (−)*	Expressed in **liver**, **lung**, **colon**, brain, kidney, skeletal muscle, spleen, and testis	No data available
*Gm16063 (+)*	Expressed in **liver**, **lung**, colon, brain, kidney, skeletal muscle, spleen, and testis	No data available
*Gm16064 (+)*	Expressed in **colon, kidney, lung, spleen,** brain, liver, skeletal muscle, and testis	No data available
*Tbx3os2 (−)*	Expressed in **kidney, lung,** brain, colon, liver, adrenal gland, and spleen	No data available
*Gm10390 (−)*	Expressed in **brain, liver, spleen, testis,** heart, skeletal muscle, colon, and lung	No data available

^a^Based on Ensembl release 74; protein coding genes are shown in bold; (+) indicates genetic element annotated on forward strand and (−) indicates reverse strand

^b^Expression documented for C57BL6 mouse strain in atlas by European Bioinformatics Institute [28]; organs of high expression are shown in bold

Among all, *Tbx3* and *Tbx5* were chosen as potential candidate genes based on their conservation between mouse and human, documented role in transforming growth factor-beta (TGF-β) [37], bone morphogenic protein (BMP) [38, 39], Hedgehog (Hh) [18], and Wnt/β-catenin [40, 41] bone signaling pathways, the link of mutations in these genes to human disorders involving bone phenotype [16, 22], and reported expression in spleen and bone of the C57BL6 mouse strain [28].

### Genetic variations in regulatory regions of the *Tbx3* and *Tbx5* genes

Sequencing of the *Tbx3* and *Tbx5* genes revealed a number of variations in regulatory and intronic regions, but none in the coding region (Table 4). Computational analysis of these variations predicted that some of these might affect promoter function, transcription factor binding, or microRNA (miRNA) binding (Table 4).

**Table 4:** Genetic variations between B10.RIII and *Eae39r2* sub-congenic mice

Genetic variations related to the mouse *Tbx3* gene				
SNP ID	Location in genome^a^	Alleles^b^	Functional class	Predicted consequence^c^
rs234834055	5:119669859	C/−	Promoter variant	May affect gene expression level
rs48522093	5:119670996	C/G	5’ UTR variant	Introduction of new TFBS for f (alpha)-f (epsilon) (undocumented role)
rs221652067	5:119674641	−/AATT	Intronic variant	No TF binding site in this region
rs48057748	5:119675826	G/T	Intronic variant	No TF binding site in this region
rs260549092	5:119677177	−/A	Intronic variant	No TF binding site in this region
rs260388829	5:119680214	C/T	Intronic variant	Disruption of TFBS for c-Rel (member of NF-ĸB TF family, linked to CIA development, and lymphoid cell growth and development [86]) and Sp1 (involved in cellular processes including growth, differentiation and apoptosis)
rs223041783	5:119681737	−/(GA)_6_	Intronic variant	No TF binding site in this region
No SNP id	5:119684553	A/−	3’ UTR variant	Lies very close to mmu-miR-511 binding site (miR-511negatively regulates growth and metastasis of tumor cells in humans [87])
rs254153782	5:119684580	A/C	3’ UTR variant	No miRNA binding site in this region
Genetic variations related to the mouse *Tbx5* gene				
SNP ID	Location in genome^a^	Alleles^b^	Functional class	Predicted consequence^c^
rs247725267	5:119834384–119,834,391	(TTCC)_2_/−	Promoter variant	May affect expression level of gene
rs46426184	5:119859957	A/G	Intron variant	No TF binding site in this region
rs222502452	5:119860635	C/T	Intron variant	No TF binding site in this region
rs32145767	5:119860705	G/A	Intron variant	No TF binding site in this region
rs242983646	5:119860887	A/C	Intron variant	Disruption of c-fos binding site (involved in cell proliferation, growth and survival pathways, including bone and immune cells [88])
rs252594029	5:119861058	T/C	Intron variant	No TF binding site in this region
rs246838998	5:119862700	A/G	Intron variant	No TF binding site in this region
rs213393759	5:119862716	G/A	Intron variant	No TF binding site in this region
rs32145773	5:119863349	T/C	Intron variant	Disruption of Sox5 binding site (linked to Th17 cell differentiation [89])
rs32146450	5:119867116	C/T	Intron variant	No TF binding site in this region
rs50869445	5:119868930	T/C	Intron variant	No TF binding site in this region
rs48190630	5:119868943	T/C	Intron variant	Introduction of new TFBS for C/EBPbeta (regulating the expression of genes involved in immune and inflammatory responses [90])
rs32147927	5:119868969	C/T	Intron variant	No TF binding site in this region
rs228127170	5:119868977	G/A	Intron variant	No TF binding site in this region
rs49608882	5:119869004	C/T	Intron variant	No TF binding site in this region
rs45644322	5:119869140	G/A	Intron variant	No TF binding site in this region
rs48943542	5:119869144	G/A	Intron variant	No TF binding site in this region
rs6373056	5:119871760	G/A	Intron variant	No TF binding site in this region
rs48054187	5:119878634	C/T	Intron variant	No TF binding site in this region
rs265584798	5:119883860	C/T	3’ UTR variant	No miRNA binding site in this region
rs255866543	5:119884274	A/G	3’ UTR variant	Lies very close to mmu-miR-6918-5p binding site (undocumented role)
rs232783988	5:119885135	−/G	3’ UTR variant	No miRNA binding site in this region
rs237950985	5: 119885225	C/T	Downstream variant	NA
rs237459586	5:119885320–119,885,324	TCTTT/−	Downstream variant	NA
rs258422458	5:119885423	C/T	Downstream variant	NA

^a^Based on Ensembl release 74 (mouse genome assembly GRCm38)

^b^Alleles are mentioned as B10.RIII/*Eae39r2*

^c^Information is based on transcription factor binding site (TFBS) prediction by ConSite [32] and PROMO [33] tools, and miRNA target prediction by miRDB [34]

*CIA* collagen-induced arthritis

### Reduced expression of *Tbx3* in B lymphocytes

**Fig. 3:**
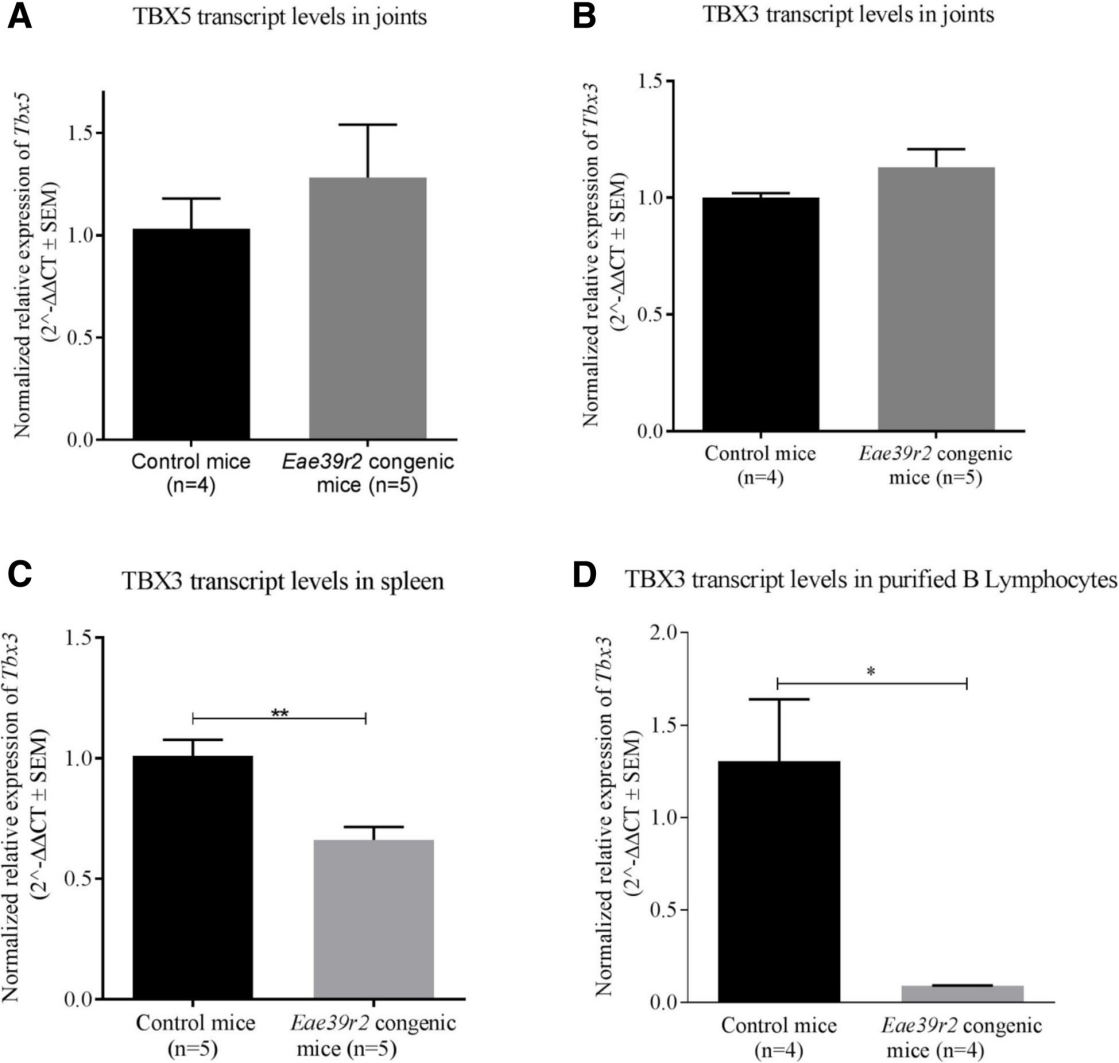
Effect of the *Eae39r2* sub-congenic fragment on *Tbx5* (**a**) and *Tbx3* (**b**) mRNA levels in the joints, *Tbx3* mRNA levels in spleen (**c**) and purified splenic B lymphocytes (**d**). Values are presented as mean of 2^^-ΔΔCt^ and bars represent standard error of the mean (SEM). Statistical analyses were performed using two-way Student’s *t* test; *p < 0.05, **p < 0.01

Since variations in the regulatory regions of the genes may affect DNA transcription, mRNA levels of *Tbx3* and *Tbx5* were measured in the spleens and joints of naïve B10.RIIIS/J-*Eae39r2* sub-congenic and littermate control mice. Both *Tbx3* and *Tbx5* were equally expressed in the joints of naïve sub-congenic and control mice (Fig. 3a, b). On the contrary, the sub-congenic mice had significantly less *Tbx3* mRNA in the spleen as compared to the control mice (*p* = 0.0036, Fig. 3c), while *Tbx5* was not detectable in the spleen.

In order to determine which cell type mainly contributed to the expression of *Tbx3* in the spleen, quantitative RT-PCR was carried out on purified B and pan T lymphocytes. We found that *Tbx3* expression was only detectable in B lymphocytes, and B10.RIIIS/J-*Eae39r2* sub-congenic mice had significantly less *Tbx3* transcripts in B lymphocytes as compared to the control mice (*p* = 0.0286, Fig. 3d).

### *TBX3* is associated with B cell proliferation but not development of B cells

**Fig. 4:**
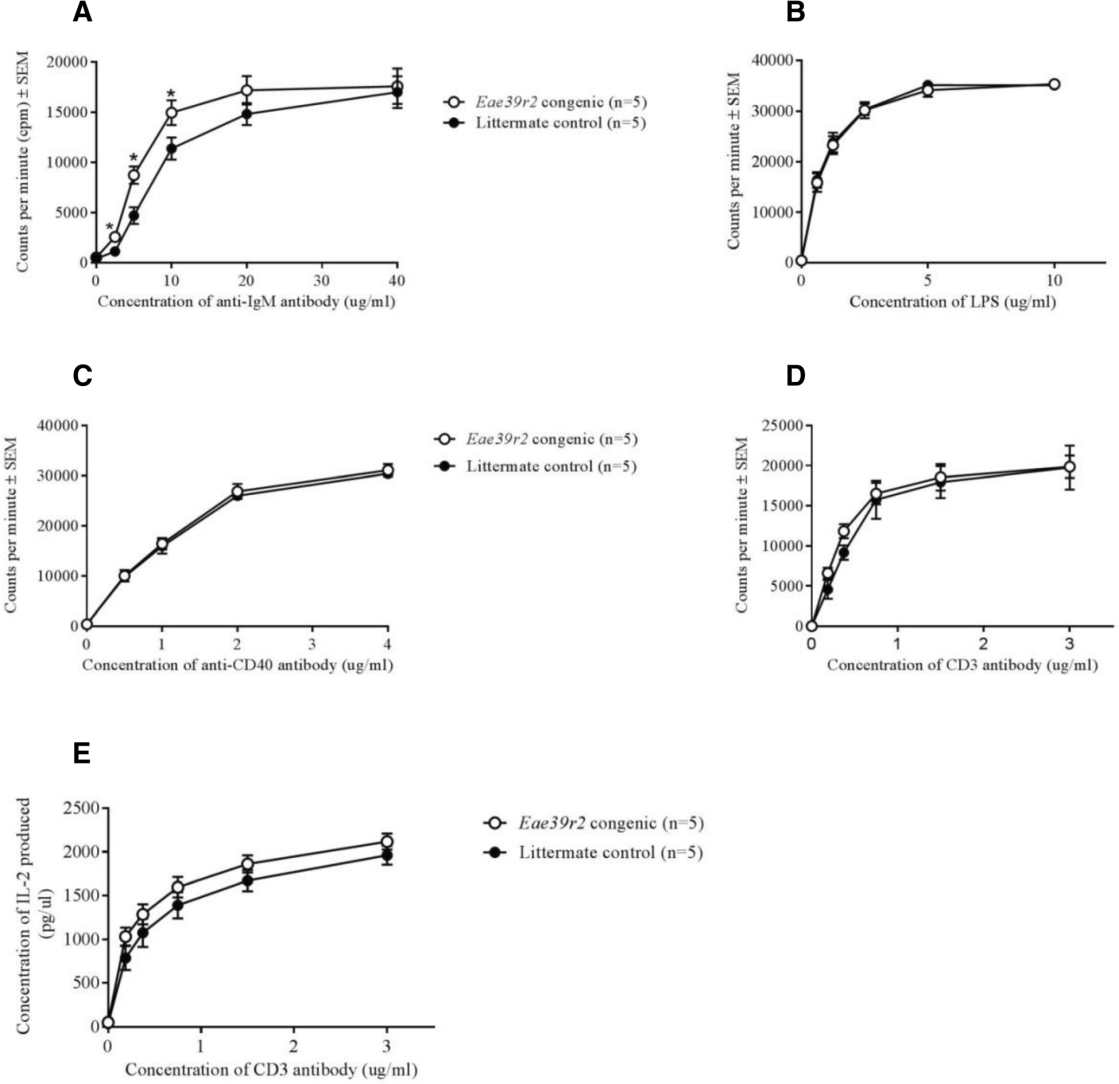
In vitro proliferative response of CD19^+^ B cells and CD4^+^ T cells from B10.RIIIS/J-*Eae39r2* sub-congenic mice. ^3^H-thymidine incorporation, represented as cpm ± SEM, of CD19^+^ B cells following in vitro stimulation with titrated concentrations (0–40 μg/ml) of anti-IgM antibody (**a**); titrated concentrations (0–10 μg/ml) of lipopolysaccharide LPS (**b**); combination of fixed concentration (10 ng/ml) of IL-4 and titrated concentrations (0–4 μg/ml) of anti-CD40 antibody (**c**). **d** ^3^H-thymidine incorporation of CD4^+^ T cells following in vitro stimulation with combination of titrated concentrations (0–3 μg/ml) of anti-CD3 antibody and fixed concentration of anti-CD28 antibody (3 μg/ml). **e** IL-2 production (mean ± SEM) in the supernatant of CD4^+^ T cells stimulated with anti-CD3/CD28 antibodies. Statistical analyses were performed using the Mann-Whitney U test; *p < 0.05

To investigate whether the observed differential expression of *Tbx3* in the *Eae39r2* sub-congenic mouse spleen was accompanied by altered immune cell activation, an in vitro proliferation assay was performed with purified CD19^+^ B cells from naïve B10.RIIIS/J-*Eae39r2* sub-congenic and control mice. B cell proliferation was significantly increased in the sub-congenic mice in response to anti-IgM stimulation (*p* = 0.0317, Fig. 4a). However, no difference in proliferation of B cells upon stimulation with either LPS or anti-CD40 antibody/IL-4 was observed (Fig. 4b-c). Furthermore, no difference in proliferation or IL-2 production by purified CD4^+^ T cells, following stimulation with anti-CD3/CD28 antibodies, was observed when comparing sub-congenic mice with littermate controls (Fig. 4d-e).

To determine whether the reduced expression of *Tbx3* had an effect at the B cell developmental level, the total and relative numbers of splenic B cells in naïve B10.RIIIS/J-*Eae39r2* sub-congenic and control mice were determined by automated cell counter and flow cytometry, respectively. No difference in the absolute number of splenic B cells (mean ± SEM, B10.RIIIS/J-*Eae39r2* sub-congenic 38.4 × 10^6^ ± 2.8 × 10^6^ (*n* = 5), control 36.9 × 10^6^ ± 3.0 × 10^6^ (*n* = 7)) and relative number of splenic B cells (mean ± SEM: B10.RIIIS/J-*Eae39r2* sub-congenic 59.9% ± 0.8 (*n* = 10), control 58.7% ± 0.5 (n = 7)) was found between the two groups of mice, indicating that TBX3 is associated with proliferation of B cells, but not the number.

**Fig. 5:**
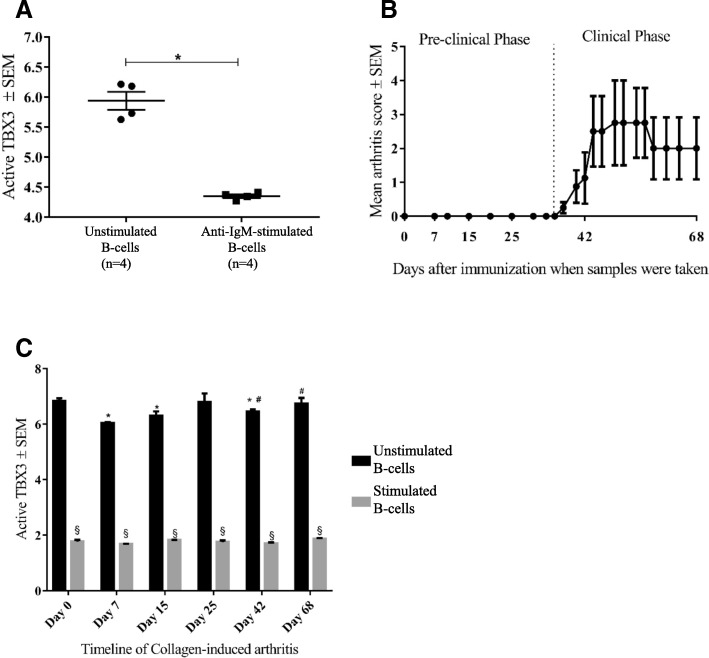
Quantitative analysis of active TBX3 in nuclear lysates of CD19^+^ B cells from B10.RIII mice. **a** Comparison of active TBX3 levels in CD19^+^ B cells isolated from naïve B10.RIII mice (*n* = 4), with and without in vitro stimulation with a fixed concentration (10 μg/ml) of anti-IgM antibody for 48 h. **b**, **c** Collagen-induced arthritis (CIA) development and corresponding TBX3 activity in B10.RIII mice whereby four mice were killed at indicated time points after collagen type II (CII) immunization and levels of active TBX3 were determined with and without ex vivo re-stimulation with fixed concentration (10 μg/ml) of anti-IgM antibody for 48 h. Statistical analyses were performed using the Mann-Whitney U test; *significant (*p* < 0.05) compared to day 0 unstimulated samples; ^#^significant compared to day 7 unstimulated samples; ^§^significant compared to respective unstimulated samples

We further analyzed changes in intra-nuclear TBX3 levels in relation to B cell activation and proliferation by comparing the quantity of active TBX3 in unstimulated B cells and in B cells activated through the BCR by anti-IgM antibody. We found that TBX3 activity in B cells was strongly downregulated upon in vitro stimulation with anti-IgM antibody (*p* = 0.0286; Fig. 5a). These data suggested that TBX3 is involved in BCR downstream pathways and might contribute to humoral immune responses.

### TBX3 activity varies in splenic B cells during the course of CIA

Based on our findings, we sought to examine the activity of TBX3 in B cells during the course of CIA. In that time-course study, mice started to show signs of arthritis on day 37 post immunization, which peaked around day 50 and slightly declined in the late phase, as shown in Fig. 5b. The overall incidence of disease was 75%, which is in accordance with previous reports on the B10.RIII mouse strain [42]. By comparing the intra-nuclear TBX3 activity in purified B cells, we found that the amount of active TBX3 falls significantly in the early pre-clinical phase (days 7 and 15) and early clinical phase (day 42) after receiving the first and booster dose of collagen, respectively (Fig. 5c). Furthermore, a significant decrease in the level of TBX3 was observed upon ex vivo re-stimulation of the B cells with anti-IgM antibody*,* at all timepoints of sampling in CIA (Fig. 5c). On the contrary, no significant variation in TBX3 activity was found in joint tissue homogenates from the same CIA time-points as for splenic B cells (data not shown).

### TBX3 - a potential biomarker of arthritis

**Fig. 6:**
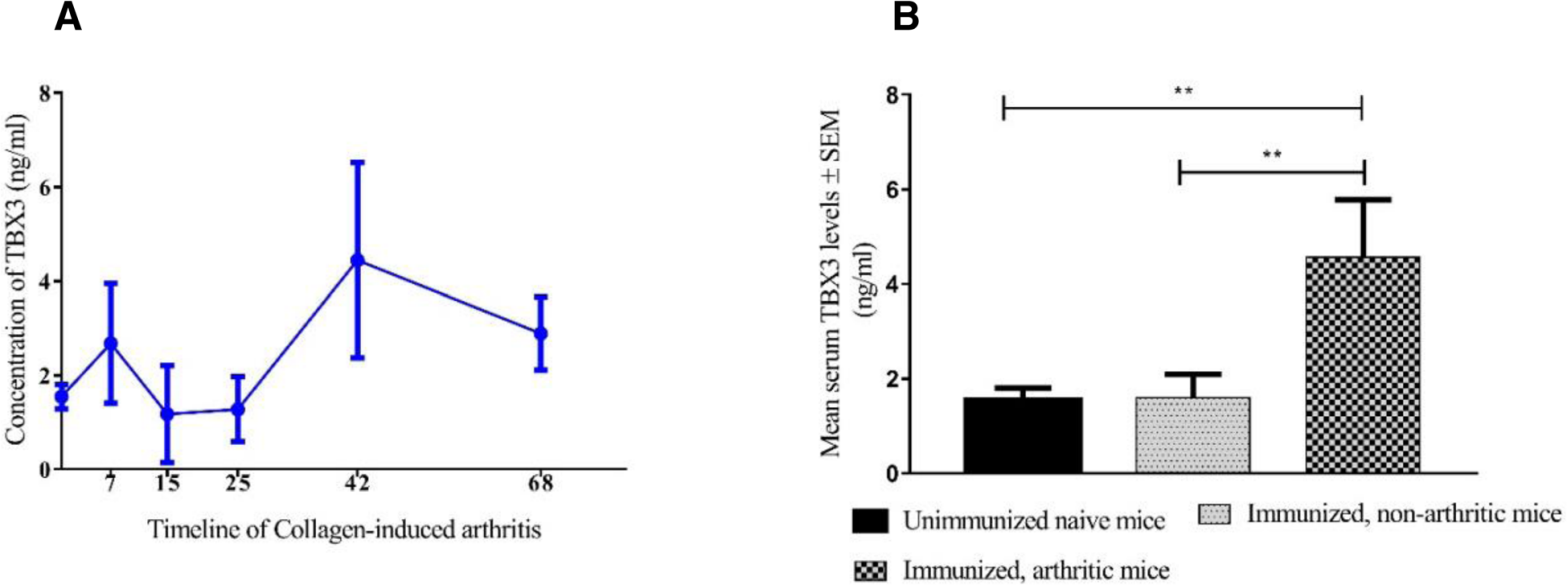
Kinetics of the expression of the TBX3 protein during development of collagen-induced arthritis (CIA) in the B10.RIII mouse strain. Levels of TBX3 in serum from CIA-immunized mice (**a**); comparison of serum-TBX3 levels between naïve mice (*n* = 4), immunized, non-arthritic mice (*n* = 14) and arthritic mice (*n* = 6) (**b**). Statistical analyses were performed using the Mann-Whitney U test; **p < 0.01

By studying the kinetics of TBX3 protein expression in serum, we made two key observations: first, TBX3 can be measured in mouse serum and second, it is dynamic along the timeline of CIA, with a considerable rise in the clinical phases (days 42 and 68) of disease in comparison to naïve and pre-clinical levels (Fig. 6a). Moreover, we showed that arthritic mice (sick mice from days 42 and 68 samples; *n* = 6) had significantly higher TBX3 levels in serum than immunized, non-arthritic mice (days 7, 15, 25, and non-arthritic mice from days 42 and 68; *n* = 14) (*p* = 0.0087) and naïve control mice (*p* = 0.0095) Fig. 6b. Spearman’s correlation coefficient was calculated to determine the relationship between the CIA clinical score and TBX3 serum levels (*r*_s_ = 0,62, *n* = 24, *p* = 0,0012). These data suggest that severity of arthritis in the CIA model is associated with an increased serum concentration of TBX3.

## Discussion

In this study, we have shown that a 1.4-Mbp locus located on mouse chromosome 5, comprising three protein coding genes and a number of non-protein coding genetic elements (Fig. 1), controls the severity of clinical arthritis in CIA along with anti-CII antibody titers. Based on a literature survey and the link to other bone diseases in humans, we selected *Tbx3* and *Tbx5*, as potential candidate genes for CIA. We demonstrated variations in the regulatory part of the *Tbx3* gene, which may affect the observed difference in expression level of *Tbx3* in splenic B lymphocytes, in addition to the increased in vitro B cell activation and proliferation upon stimulation through cell-bound IgM. Moreover, our functional data showed that TBX3 activity is inversely related to B cell activation both in vitro and in the CIA model. This suggests that TBX3 is a player in CIA pathogenesis by modulating B cell proliferative responses, but that it does not affect the development of B lymphocytes.

Identification of quantitative trait loci (QTLs), genetic fine-mapping by using congenic and sub-congenic mice, and the discovery of novel candidate genes, have led to the identification of molecular interactions that underlie complex diseases [43–45]. In this study, we fine-mapped a previously reported QTL, *Eae39*, linked to CIA [8, 10]. By breeding of sub-congenic lines and by using the same immunization protocol, we have shown that the *Eae39* region contains a smaller QTL (*Eae39r*), which encompasses 1.8 Mbp and has an enhancing effect on CIA severity. The genes within the fragment are *Med13l*, *Tbx3*, *Tbx5*, and *Rbm19*, coding for the mediator complex subunit 13-like protein, the T-box protein 3, T-box protein 5, and RNA binding motif protein 19, respectively. CIA experiments with sub-congenic mice provide an attractive opportunity to study the individual (or combined) contribution of these genes towards arthritis development. We have previously shown that B10.RIIIS/J-*Eae39r1* sub-congenic mice (with only *Med13l* in the sub-congenic fragment) develop arthritis to the same extent as the wild-type B10.RIII mice [46]. In the current study, we showed that B10.RIIIS/J-*Eae39r2* sub-congenic mice (with *Tbx3*, *Tbx5* and *Rbm19* in the sub-congenic fragment) develop more severe arthritis compared to the control mice. Taken together, it is reasonable to conclude that the CIA-enhancing effect seen in the B10.RIIIS/J-*Eae39r* mice is due to the genes located in the *Eae39r2* fragment and that MED13L has minimal or no effect on CIA development. Additionally, the *Eae39r2* region controls the anti-CII antibody response.

Within the *Eae39r2* locus, two evolutionarily related genes, *Tbx3* and *Tbx5*, were chosen as potential candidate genes controlling CIA severity based on the consensus criterion recommended by the Complex Trait Consortium [47]. We cannot, however, disregard the involvement of non-coding genes within *Eae39r2*. In this study we have reported a number of polymorphisms in regulatory regions of the *Tbx3* and *Tbx5* genes when comparing the B10.RIII and RIIIS/J parental mouse strains. Some of the polymorphisms are predicted to affect the transcription and protein expression levels by modulating promoter activity, transcription factor binding, or microRNA binding. We propose that one or more of these variants are responsible for the allele-specific differences in *Tbx3* transcript levels observed in total spleen and splenic B lymphocytes, but not in joints. This can be explained by tissue-specific regulation of *Tbx3* that has been previously documented in mice [18] and humans [18, 25].

It has recently been acknowledged that B lymphocytes and autoantibodies are key in aspects of RA pathogenesis [48, 49] and the clinical success of B cell targeted therapies further strengthens this [6, 50]. Although not directly demonstrated, we believe that decreased expression of *Tbx3* in B lymphocytes from mice with a 1.4-Mbp fragment derived from a genetically different mouse strain, in addition to the increased in vitro proliferation of these cells in response to anti-IgM stimulation, is a finding that can explain the enhanced antibody response and more severe disease in these mice. We suggest that the increased proliferation of B cells from the B10.RIIIS/J-*Eae39r2* sub-congenic mice in response to anti-IgM stimulation, but not to LPS or anti-CD40/IL-4 treatment, might be the result of TBX3 interacting with specific components of the intracellular pathways activated. Anti-IgM antibodies stimulate B cells through the B cell receptor (BCR) thereby activating further downstream signaling cascades like the phosphatidylinositol 3-kinase (PI3K), protein kinase B (AKT) and mitogen-activated protein kinase (MAPK) pathways [51–53]. TBX3 has previously been shown to directly interact with these signaling cascades in other cell types to regulate cell survival and proliferation [54–57] and a similar mechanism might operate in B cells.

The importance of TBX3 in relation to proliferation is underpinned by studies of its role in oncogenesis [24, 25, 58, 59]. TBX3 functions primarily as a transcriptional repressor and initial reports suggested that it can promote proliferation by repressing the cell cycle regulators p14/p19^ARF^ and p21 [60–62]. More recently, it was recognized that although TBX3 promotes migration and invasiveness of cancer cells, it negatively regulates cell proliferation [37, 63]. In line with these reports of an anti-proliferative role for TBX3, our study suggests that it might be controlling cell cycle regulators in B lymphocytes under normal conditions. Lack of, or low expression of *Tbx3*, may thus lead to increased activation of B lymphocytes, manifested by proliferation and increased autoantibody production, which subsequently would cause severe arthritis in the CIA model. To this end, we demonstrated that the level of functionally active TBX3 in B cells falls after in vitro activation through the BCR. Furthermore, our in vivo results from the time-course CIA study show that TBX3 activity falls significantly when the immune system is challenged through heterologous CII immunization. The lowest TBX3 activity was observed a week after the first injection of immunogen, which corresponds to the time point reported for the highest B cell activation and proliferation of CII-specific IgM-positive B cells in the CIA model [64]. This, again, suggests that TBX3 interacts with signaling pathways downstream of the IgM-BCR, thereby being modulated by, or modulating, the activation signal, resulting in consequences for the outcome of the B cell response.

To our knowledge, this is the first study to suggest the involvement of TBX3 in B cell proliferation and development of CIA, and adds mechanistic links to three independent genome-wide association studies (GWAS) that linked *TBX3* to RA [65–67]. Whether the TBX3 genetic variant(s) linked to RA in these studies represents a true susceptibility variant(s) is still to be investigated. Furthermore, our preliminary data show that TBX3 is a putative RA biomarker and that an increase in TBX3 serum levels in untreated arthritis reflects dysregulation of currently unidentified disease pathways. Although the current biomarker study has limitations in terms of group size and that the studies are performed in an experimental model, the conservation of this protein and disease pathways between man and mouse might overcome the discovery step, which is often a bottleneck in the biomarker field. Future studies in human cohorts are needed to prove the biomarker potential of the multifaceted protein, TBX3.

## Conclusion

We have fine-mapped the CIA enhancing *Eae39r* locus and suggest that the therein located *Tbx3* gene is a disease candidate gene for experimental arthritis in the mouse*.* From studies of gene transcription and B cell proliferation, we propose that TBX3 impacts CIA development and autoantibody production by regulating B lymphocyte activation. Taken together, these results indicate that TBX3 is involved in RA pathogenesis and possibly also in other autoimmune diseases dependent on B cell activation and autoantibody production. By investigating intracellular TBX3 protein activity in B cells upon stimulation through the B cell receptor, we have shown that activation of B cells is concomitant with low levels of active TBX3 in vitro. Moreover, we observed that serum levels of TBX3 follow arthritis severity. We therefore propose TBX3 as a candidate biomarker that can potentially aid in diagnosis and assessment of RA disease severity.

## Additional file

Additional file 1: **Table S1.** List of primers used for genotyping of mice. **Table S2.** List of primers for *Tbx3* and *Tbx5* sequencing. **Table S3.** List of primers used for real time PCR. (DOCX 22 kb)

## Abbreviations

BCR: B cell receptor
bp: Base pairs
BSA: Bovine serum albumin
CIA: Collagen-induced arthritis
CII: Collagen type II
DMEM: Dulbecco’s modified Eagle’s medium
ELISA: Enzyme-linked immunosorbent assay
FBS: Fetal bovine serum
FCS: Fetal calf serum
GWAS: Genome-wide association studies
IFA: Incomplete Freund’s adjuvant
IL: Interleukin
LPS: Lipopolysaccharide
Mbp: Mega base pair
MHC: Major histocompatibility complex
PBS: Phosphate-buffered saline
QTL: Quantitative trait locus
RA: Rheumatoid Arthritis
SNP: Single nucleotide polymorphism
TBX3: T-box protein 3
TFBS: Transcription factor binding site
UTR: Untranslated region
