# Supplementary material for: The oncoprotein TBX3 is controlling severity in experimental arthritis
Source: Arthritis Res Ther. 2019 Jan 10;21:16. doi: 10.1186/s13075-018-1797-3 (PMC6329118; doi:10.1186/s13075-018-1797-3)
Supplement: Supplementary file 1 — Table S1. List of primers used for genotyping of mice. Table S2. List of primers for Tbx3 and Tbx5 sequencing. Table S3. List of primers used for real time PCR. (DOCX 22 kb) [file 13075_2018_1797_MOESM1_ESM.docx]

**Additional file**

**Table S1**. List of primers used for genotyping of mice

| **Primers for genotyping** | | | |
| --- | --- | --- | --- |
| **SNP/Marker id** | **Location** | **Primer Sequences** | **Annealing Temperature** |
| rs33583463 | 5:118596773 | FP: 5' GCTGCCTCGCTACATGGTAT 3'  RP: 5' GGGCTCCAGATACTTCAAGC 3' | 63°C |
| rs29824716 | 5:120043597 | FP: 5' TCAGGAGAACAACCCTCAGC 3'  RP: 5' GACAAACCCCTCAAATCCAA 3' | 65°C |
| D5tbxhm17 | 5:119660373 | FP: 5´CACCTTGGCTCTTGAGGTATTCT 3’  RP: 5´GCTCTGAGACTCCACCTGTTCTA 3´ | 63°C |

FP: forward primer, RP: reverse primer

**Table S2**. List of primers for *Tbx3* and *Tbx5* sequencing

| **SNP ID** | **Location** | **Primer Sequences** | **Annealing Temperature** |
| --- | --- | --- | --- |
| rs234834055 | 5:119669859 | FP: 5´ ACCCAACCAAAGTCTGATCG 3´  RP: 5´ GATCCCACCAATCTTTCTGC 3´ | 60°C |
| rs48522093 | 5:119670996 | FP: 5´ ACCCAACCAAAGTCTGATCG 3´  RP: 5´ GATCCCACCAATCTTTCTGC 3´ | 60°C |
| rs221652067 | 5:119674641 | FP: 5´ CCTGGGTATGGAAAGCTTGA 3´  RP: 5´ CTTTCACAGGCAACCCAAGT 3´ | 60°C |
| rs48057748 | 5:119675826 | FP: 5´ TCCCGGAAACAGAATTCATC 3´  RP: 5´ TCTCTGGCTATGAGGGGAGA 3´ | 62°C |
| rs260549092 | 5:119677177 | FP: 5´ TGGTAAGGAGCAGGAAGTGG 3´  RP: 5´ CATTGCCAGTGTCTCGAAAA 3´ | 60°C |
| rs260388829 | 5:119680214 | FP: 5´ AGACAATAAACCGCCCAGTG 3´  RP: 5´ GAGTCTGGCGATGCCTTGT 3´ | 62°C |
| rs223041783 | 5:119681737 | FP: 5´ CCTTTCTCTTCCACCCGACT 3´  RP: 5´ GCAGAGAGGTAGGGTTGGTG 3´ | 60°C |
| No SNP id | 5:119684553 | FP: 5´ CCAGGTCAGGAGATGGCTAA 3´  RP: 5´ TCCGGCTCTGAAAGACAACT 3´ | 60°C |
| rs254153782 | 5:119684580 | FP: 5´ CCAGGTCAGGAGATGGCTAA 3´  RP: 5´ TCCGGCTCTGAAAGACAACT 3´ | 60°C |
| rs247725267 | 5:119834384-119834391 | FP: 5´ CTGGGCACTGCTCTGTGTA 3´  RP: 5´ CCACCTTTCCCACTTTCTCA 3´ | 60°C |
| rs46426184 | 5:119859957 | FP: 5´ AGCATTGCCTGGGAGTCTTA 3´  RP: 5´ TCCTCTCCACCTCTGTCACC 3´ | 60°C |
| rs222502452 | 5:119860635 | FP: 5´ CATGGGGACAACTTCGTGA 3´  RP: 5´ TGCTGGGATTAAAGGTCCAC 3´ | 60°C |
| rs32145767 | 5:119860705 | FP: 5´ CATGGGGACAACTTCGTGA 3´  RP: 5´ TGCTGGGATTAAAGGTCCAC 3´ | 63°C |
| rs242983646 | 5:119860887 | FP: 5´ CATGGGGACAACTTCGTGA 3´  RP: 5´ TGCTGGGATTAAAGGTCCAC 3´ | 63°C |
| rs252594029 | 5:119861058 | FP: 5´ CATGGGGACAACTTCGTGA 3´  RP: 5´ TGCTGGGATTAAAGGTCCAC 3´ | 60°C |
| rs246838998 | 5:119862700 | FP: 5´ TCGAAGGAACTCTGCGATGT 3´  RP: 5´ ACGAACAGTCAACAGGTCCA 3´ | 60°C |
| rs213393759 | 5:119862716 | FP: 5´ TCGAAGGAACTCTGCGATGT 3´  RP: 5´ ACGAACAGTCAACAGGTCCA 3´ | 60°C |
| rs32145773 | 5:119863349 | FP: 5´ AGCTTCCTTGGGACAAGTTCT 3´  RP: 5´ GGCTTGTGGGTGACTTTGAT 3´ | 63°C |
| rs32146450 | 5:119867116 | FP: 5´ CTGCAGTGTAGAGGGAAGGT 3´  RP: 5´ ACCAGACACAAGCCAGATCT 3´ | 60°C |
| rs50869445 | 5:119868930 | FP: 5´ TTCTGGCTGCATCTTGACC 3´  RP: 5´ TGGATGTGGCTCAGTAGCA 3´ | 60°C |
| rs48190630 | 5:119868943 | FP: 5´ TTCTGGCTGCATCTTGACC 3´  RP: 5´ TGGATGTGGCTCAGTAGCA 3´ | 60°C |
| rs32147927 | 5:119868969 | FP: 5´ TTCTGGCTGCATCTTGACC 3´  RP: 5´ TGGATGTGGCTCAGTAGCA 3´ | 63°C |
| rs228127170 | 5:119868977 | FP: 5´ TTCTGGCTGCATCTTGACC 3´  RP: 5´ TGGATGTGGCTCAGTAGCA 3´ | 60°C |
| rs49608882 | 5:119869004 | FP: 5´ TTCTGGCTGCATCTTGACC 3´  RP: 5´ TGGATGTGGCTCAGTAGCA 3´ | 60°C |
| rs45644322 | 5:119869140 | FP: 5´ TTCTGGCTGCATCTTGACC 3´  RP: 5´ TGGATGTGGCTCAGTAGCA 3´ | 60°C |
| rs48943542 | 5:119869144 | FP: 5´ TTCTGGCTGCATCTTGACC 3´  RP: 5´ TGGATGTGGCTCAGTAGCA 3´ | 62°C |
| rs6373056 | 5:119871760 | FP: 5´ CAGTTCAGCTTCTCCCTGGA 3´  RP: 5´ TGTGTGTTATGTATGCGCATG 3´ | 60°C |
| rs48054187 | 5:119878634 | FP: 5´ TCCAGTGGGCAGAAGGAAAT 3´  RP: 5´ CACACAGTGCCAACCATACA 3´ | 60°C |
| rs265584798 | 5:119883860 | FP: 5´ GTGTCGAGTCCCATCCTACC 3´  RP: 5´ AGGTTTCCAGGCTGAGGAGT 3´ | 60°C |
| rs255866543 | 5:119884274 | FP: 5´ GTGTCGAGTCCCATCCTACC 3´  RP: 5´ AGGTTTCCAGGCTGAGGAGT 3´ | 63°C |
| rs232783988 | 5:119885135 | FP: 5´ GTGTCGAGTCCCATCCTACC 3´  RP: 5´ AGGTTTCCAGGCTGAGGAGT 3´ | 60°C |
| rs237950985 | 5: 119885225 | FP: 5´ TATGTGGCTGAGAACGTGGA 3´  RP: 5´ TTTCCTGCTCCACTCTGCTT 3´ | 60°C |
| rs237459586 | 5:119885320-119885324 | FP: 5´ TATGTGGCTGAGAACGTGGA 3´  RP: 5´ TTTCCTGCTCCACTCTGCTT 3´ | 60°C |
| rs258422458 | 5:119885423 | FP: 5´ TATGTGGCTGAGAACGTGGA 3´  RP: 5´ TTTCCTGCTCCACTCTGCTT 3´ | 63°C |

FP: forward primer, RP: reverse primer

**Table S3**. List of primers used for real time PCR

| **Primers for RT-PCR** | | | |
| --- | --- | --- | --- |
| **Gene** | **Transcript id** | **Primer Sequences** | **Annealing Temperature** |
| *Tbx3* | 001, 201, 002 | FP: 5´ CCACCCGTTCCTCAATTTGAACAG 3´  RP: 5´CGGAAGCCATTGATGGTAAAGCTG 3´ | 63°C |
| *Tbx5* | 201 | FP: 5´ TCCGGCTTTCCTGCTAAGA 3´  RP: 5´ GGCCAAAGCCCTCATCTGTAT 3´ | 60°C |

FP: forward primer, RP: reverse primer
